# Evaluation of genetic variability among “Early Mature” *Juglans regia* using microsatellite markers and morphological traits

**DOI:** 10.7717/peerj.3834

**Published:** 2017-10-26

**Authors:** Aziz Ebrahimi, Abdolkarim Zarei, Mojtaba Zamani Fardadonbeh, Shaneka Lawson

**Affiliations:** 1 Department of Forestry and Natural Resources, Purdue University, West Lafayette, IN, United States of America; 2 Department of Biotechnology, Jahrom University, Jahrom, Fars, Iran; 3 Department of Agronomy and Plant Biotechnology, University of Tehran, Karaj, Alborz, Iran; 4 USDA Forest Service, Hardwood Tree Improvement and Regeneration Center (HTIRC), Purdue University, West Lafayette, IN, United States of America

**Keywords:** Persian walnut, SSR markers, Genetic diversity, “Early Mature”, Cluster analysis

## Abstract

Limiting the juvenile phase and reducing tree size are the two main challenges for breeders to improve most fruit crops. Early maturation and dwarf cultivars have been reported for many fruit species. “Early mature” and low vigor walnut genotypes were found among seedlings of Persian walnut. Nine microsatellite markers were used to evaluate genetic diversity among “Early Mature” Persian walnut accessions and provide a comparison with “normal growth” accessions. Six maturation related characteristics were also measured in “Early Mature” samples. Phenotypic traits and diversity indices showed relatively high levels of genetic diversity in “Early Mature” seedlings and indicated high differentiation between individuals. Seedling height, the most diverse phenotypic trait, has an important role in the clustering of “Early Mature” accessions. The “Early Mature” type had higher number of alleles, number of effective allele, and Shannon index compared to the “Normal Growth” group. The two types of studied walnuts had different alleles, with more than half of produced alleles specific to a specific group. “Early Mature” and “Normal Growth” walnuts had 27 and 17 private alleles, respectively. Grouping with different methods separated “Early Mature” and “Normal Growth” samples entirely. The presence of moderate to high genetic diversity in “Early Mature” walnuts and high genetic differentiation with “Normal Growth” walnuts, indicated that “Early Mature” walnuts were more diverse and distinct from “Normal Growth” samples. Moreover, our results showed SSR markers were useful for differentiating between “Early Mature” and “Normal Growth” walnuts. A number of identified loci have potential in breeding programs for identification of “Early Mature” walnuts at the germination phase.

## Introduction

Early maturation is an important attribute in fruit and nut species. This characteristic has great economic value due to accelerated returns on investments and use of higher intensity planting systems than normal sized trees. A primary constraint in tree breeding is the extended juvenile phase, wherein the tree limits productivity to vegetative growth before sufficient vigor is achieved for the reproductive phase. Juvenile phase length is species dependent and varies in fruit and nut species from 3–4 years in almond, peach, and cherry; 5–10 years in *Citrus* fruits, apple, pear, pistachio and walnut; to more than 15 years in avocado and date palm (Nocker & Gardiner, 2014). Shortening the juvenile period is an important objective for nut orchard management and breeding programs, but more research is needed to evaluate “Early Mature” traits. Several methodologies can help facilitate this objective. These include manipulating culture conditions to promote vigorous growth, use of biotechnological approaches such as promoting flowering gene expression (LEAFY and APETALA), suppression expression of delayed flowering gene (TERMINAL FLOWER or TFL1), and using natural diversity from different gene pools (Hanke et al., 2007).

Persian walnut is one of the most important nut trees and has been cultivated primarily for nut production since ancient times. It is believed that this nut tree originated from Persia (Iran plateau) before diversification to other regions (McGranahan et al., 1998; Bayazit et al., 2007; Ebrahimi, Vahdati & Fallahi, 2007). Thus, Iran is rich in Persian walnut germplasm. In addition, Persian walnut is allogamous and has been propagated extensively by seed thus exhibiting a wide range of phenotypical variation among different accessions for utilization in breeding programs.

Early maturation in Persian walnut is a primary objective for walnut breeders and growers. Some accessions naturally exhibit this trait and have been identified by some Persian walnut producers, including those in the Central Asian Republics of the former Soviet Union (Germain, Delort & Kanivets, 1997), France (Breton et al., 2004), and Iran (Vahdati, Hassani & Rezaee, 2014). These “Early Mature” walnuts can flower within one year of seed cultivation compared to the typical 8–15 year juvenile period for Persian walnut seedlings. “Early Mature” walnuts also demonstrate other coveted attributes including low vegetative growth and dwarf growth habit, lateral fruit bearing, high productivity, clustered inflorescences and winter cold hardiness (Germain, Delort & Kanivets, 1997). However, due to early bud breakage, some of these walnut genotypes are susceptible to late spring frosts and walnut blight disease (Germain, Delort & Kanivets, 1997).

Microsatellites, or simple sequence repeats (SSRs), are versatile molecular markers used extensively in plant genetic studies due to their high reproducibility, co-dominance, and highly polymorphic nature. The present study evaluated several “Early Mature” walnut accessions from Iran using SSRs and phenotypic data to elucidate more about the genetic basis of these walnut types. Additionally, we used some of the “Normal Growth” walnut samples for comparison with “Early Mature” accessions at the DNA level. Ninety-three “Early Mature” walnut seedlings were evaluated in parallel with 10 “Normal Growth” accessions using SSR molecular markers.

## Materials and Methods

### Plant materials

**Figure 1:**
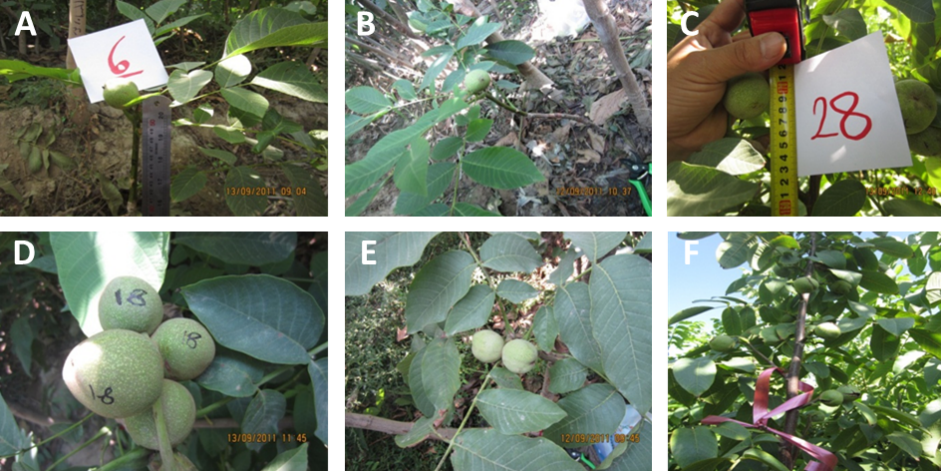
Nuts observed on the “Early Mature” Persian walnut seedlings obtained in the third year of cultivation.

Over five thousand seeds were harvested from superior quality adult walnut trees grown in Qazvin, Iran. The mother plants were originally collected from several locations of Iran. The seeds were planted in a nursery in Karaj, Iran to prepare the seedlings for sale to nut growers. Ninety-three seedlings demonstrated precocity, flowering from one to three years after cultivation, and were selected for further study (Table S1, Fig. 1). Ten typical adult walnut accessions from the Karaj condition were included in our experiment as genetic controls for “Early Mature” seedlings.

### Evaluation of maturation related attributes

Several phenotypical traits were measured in “Early Mature” accessions, including diameter and height, number of nodes, internode length, number and average weight of nuts. Traits were recorded the third year after seed germination.

### Genomic DNA extraction, PCR amplification and PCR product analysis

Genomic DNA was extracted from leaf samples using the CTAB (cetyltrimethylammonium bromide) method (Doyle & Doyle, 1987). Nine SSR primer pairs (WGA1, WGA9, WGA27, WGA32, WGA89, WGA118, WGA202, WGA276 and WGA321), originally described by Dangl et al. (2005); were chosen based on product consistency, ease of scoring, and high polymorphism rates reported in previous research (Pollegioni et al., 2009). PCR was performed according the protocols described by Ebrahimi, Fatahi & Zamani (2011) in a final reaction volume of 20 µl. Reactions were performed in a GENEAmp 9700 thermal cycler according to the following procedure: an initial denaturation at 94°C for 5 min, followed by 35 cycles of 45 s at 94°C, 45 s at the optimum annealing temperature for each primer pair, and 1 min at 72°C; then a final extension step at 72°C for 7 min.

### Statistical analysis

Descriptive statistical analyses of morphological traits were conducted using SPSS v.19 software (SPSS Inc.; Norusis, 1998). NTsys v.2.2 software was used to perform cluster analyses using the Unweighted Pair Group Method with Arithmetic Mean (UPGMA) based on Euclidean distance coefficients for accession pairs (Rohlf, 2000). The Mantel test was performed by NTsys v.2.2 software to estimate correlation between morphological and SSRs markers.

Genetic diversity parameters, such as total number of observed alleles (N_A_), observed (H_O_) and expected (H_E_) heterozygosity, effective number of alleles (A_E_), and Shannon’s information index (*I*) were computed for each locus individually and for all loci using POPGENE software v.1.31 (Yeh et al., 1997). Polymorphic information content (PIC) was estimated using CERVUS v.2.0 software (Marshall et al., 1998). GeneAlex software v.6.5 was used to perform Analysis of Molecular Variance (AMOVA), Principal Coordinate Analysis (PCA) and estimation of private alleles and rare alleles (Peakall & Smouse, 2012). Nei’s unbiased genetic distance (Nei, 1972) was used to estimate genetic distance between accessions, and a dendrogram was drawn using Mega v.6 software (Tamura et al., 2012).

STRUCTURE v.2.3.4 was used to analyze the genetic structure of the Persian walnut germplasm. The project was run with the following parameters: run length as 150,000 burnin period lengths and 250,000 Markov Chain Monte Carlo (MCMC) repetitions where *K* has values ranging from 1 to 10 and each *K* was run 10 times. The optimum value of *K* was determined by calculating the Δ*K* value to estimate the most likely number of groups (Evanno, Regnaut & Goudet, 2005). STRUCTURE results were processed with the software STRUCTURE HARVESTER v.0.6.1 (Earl & VonHoldt, 2012) to obtain the most likely *K* value.

## Results

### Phenotypic variation

**Figure 2:**
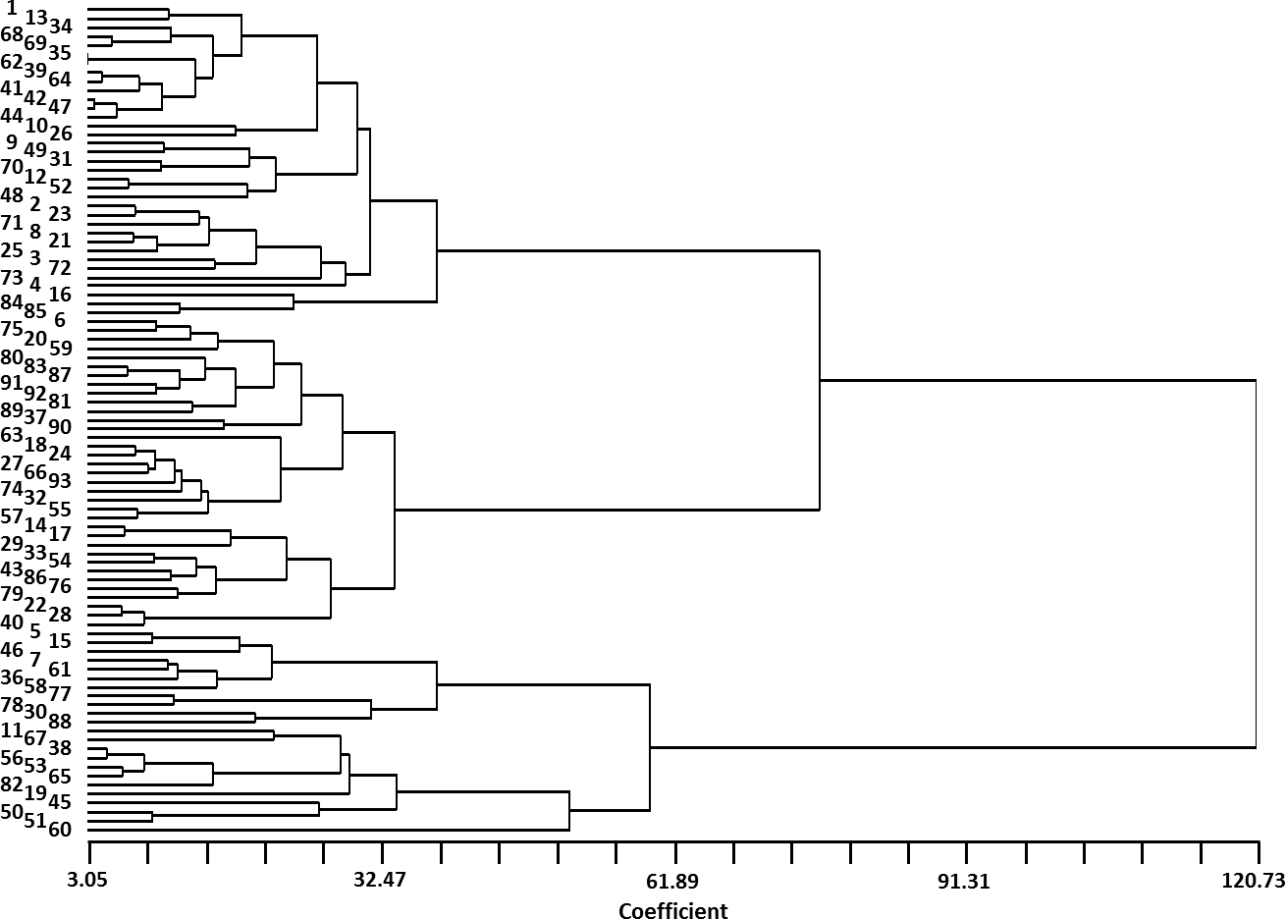
Cluster analysis of 93 “Early Mature” *J. regia* accessions based on several early-maturation phenotypic traits. Numbers on left in direct alignment with referenced accession. Accession numbers and phenotypic traits listed in Table S1.

There were significant variations among “Early Mature” traits regarding vegetative growth and nut yield (Table S1). Mean values, standard deviation (SD), and coefficients of variation (CV) values for the accessions are presented in Table 1. Each of the traits examined had CV values greater than 30%, indicating high variation among “Early Mature” accessions based on the characters investigated. The lowest CV observed was for average nut weight (CV = 30.95%), while the highest value was recorded for seedling height (CV = 72.31%). Seedling height varied from 10 cm to 240 cm with a mean of 85.83 cm. Average number of nuts was 9.35 but had high variation (1 to 29 nuts) among seedlings. Nut weight was the least variable trait studied and ranged from 1.70 g to 14.42 g (average = 7.56 g). UPGMA cluster analysis, based on morphological attributes, sorted the 93 “Early Mature” accessions into two main clusters (Fig. 2). The largest cluster was composed of 70 accessions, all of which had less vegetative growth than the second group. The second cluster was composed of 23 accessions, each with greater vegetative growth and heights greater than 100 cm in the third year following seed cultivation. Seedling height was the main factor affecting clustering.

**Table 1:** Descriptive statistics for measured traits among 93 accessions of “Early Mature” *Juglans regia*.

	Character	Abbreviation	Unit	Min.	Max.	Mean	SE	SD	CV%^a^
1	Seedling diameter	SD	mm	12	72	42.91	1.58	15.21	35.45
2	Seedling height	SH	cm	10	240	85.83	6.44	62.06	72.31
3	Number of node	NoN	–	0	40	16.15	0.96	9.25	57.28
4	Internode length	IL	cm	0	21	7.03	0.32	3.12	44.38
5	Number of nut	NoNu	–	1	29	9.35	0.69	6.67	71.34
6	Nut weight	NW	g	1.7	14.42	7.56	0.24	2.34	30.95

**Notes.**

a Coefficient of variation percentage.

### Genetic diversity

All studied loci produced polymorphic fragments in the *J. regia* samples studied. A total of 62 alleles were recorded with an average of 6.89 alleles per locus (Table 2). WGA202 had the highest number of alleles (N_A_ = 11) while WGA9, WGA27, and WGA89 had the lowest (A = 4). Eight rare alleles were observed in five loci and the WGA32 locus had the highest (A_RA_ = 3). The effective number of alleles (A_E_) ranged from 1.73 (WGA9) to 5.51 (WGA321) with a mean of 3.56. The average of polymorphic information content (PIC) was 0.65 (SE = 0.12). WGA321 had the greatest PIC value (0.82) while WGA9 displayed the lowest value (0.43). The H_O_ varied from 0.34 (WGA9) to 0.74 (WGA89 and WGA276), while H_E_ ranged from 0.42 (WGA9) to 0.83 (WGA89). The mean value of Shannon’s information index (*I*) was 1.41 and varied across loci from 0.81 (WGA9) to 1.80 (WGA202) (SE = 0.41).

**Table 2:** Information pertaining to the nine SSR loci used in this study and some of diversity indices obtained in 103 *Juglans regia* accessions.

Locus	N	Repeat type	SR (bp)	A	A_E_	PIC	*I*	H_O_	H_E_	HW	A_RA_	Ap M	Ap N
WGA1	103	(GA)5	182–192	5	3.11	0.62	1.28	0.6	0.68	NS	0	2	2
WGA9	103	(GA)16	233–247	4	1.73	0.43	0.81	0.34	0.42	NS	0	0	0
WGA27	103	(GA)30	205–215	4	2.91	0.59	1.13	0.61	0.66	*	1	2	0
WGA32	103	(CT)19	157–208	10	4.32	0.75	1.78	0.58	0.77	NS	3	6	3
WGA89	103	(GA)4	190–221	4	2.74	0.57	1.09	0.74	0.64	**	1	1	0
WGA118	103	(GA)18	178–293	8	2.97	0.61	1.31	0.63	0.67	NS	2	3	3
WGA202	103	(GA)20	198–295	11	4.03	0.73	1.8	0.58	0.76	*	1	6	4
WGA276	103	(GA)14	160–193	8	5.17	0.8	1.77	0.74	0.83	NS	0	3	2
WGA321	103	(GA)14	224–247	8	5.51	0.82	1.76	0.63	0.82	NS	0	4	3
Mean				6.89	3.56	0.65	1.41	0.61	0.69	–	0.89	3	1.89
SE				2.71	1.16	0.12	0.37	0.12	0.12		1.05	2.06	1.53

**Notes.**

N accession size SR allele size range (bp) A number obtained alleles A_E_ number effective alleles PIC polymorphic information content HW Hardy–Weinberg exact test (*p* = 0.01) *I* Shannon’s information index H_O_ observed heterozygosity H_E_ expected heterozygosity HW Hardy–Weinberg equilibrium NS not significant A_RA_ number rare alleles Ap M number private alleles (“Early Mature” samples) Ap N number private alleles (“Normal Growth” samples) SE standard error

Differences between N_A_, A_E_, and *I* were observed in “Early Mature” and “Normal Growth” *J. regia* (Table 3). Values of observed and expected heterozygosities (H_O_, H_E_) were not significantly different between the two sample groups. Several private alleles were observed in “Early Mature” and normal walnut accessions. Most studied loci had private alleles with the greatest number (6 alleles) observed in WGA32 and WGA202 loci from “Early Mature” samples. “Early Mature” walnuts had 27 private alleles, while “Normal Growth” individuals had 17 alleles.

**Table 3:** Genetic diversity of “Early Mature” and “Normal Growth” *Juglans regia* accessions using SSR loci.

Walnut type	N	A	A_E_	*I*	H_O_	H_E_	Nei	A_PA_
Early Mature	93	5.00 ± 1.41	3.25 ± 0.99	1.28 ± 0.34	0.60 ± 0.13	0.66 ± 0.13	0.66 ± 0.13	27
Normal	10	3.88 ± 1.05	2.89 ± 0.71	1.15 ± 0.26	0.67 ± 0.19	0.67 ± 0.09	0.64 ± 0.09	17
Mean ± SD	51.50 ± 58.69	4.44 ± 0.79	3.07 ± 0.26	1.22 ± 0.09	0.64 ± 0.05	0.67 ± 0.001	0.65 ± 0.01	22 ± 7.07

**Notes.**

N accession size A number obtained alleles A_E_ number effective alleles *I* Shannon’s information index H_O_ observed heterozygosity H_E_ expected heterozygosity Nei Nei’s genetic distance A_PA_ number private alleles (“Normal Growth” samples) SE standard error

### Genetic structure of germplasm

Cluster analysis based on Nei’s genetic distance matrix using the Neighbor-joining method divided the accessions into two main groups (Fig. S1). “Normal Growth” walnuts were separated from “Early Mature” accessions that were subsequently divided into seven sub-groups. Accession number 40 diverged from other “Early Mature” walnuts and formed a distinct sub-group. The largest sub-group (VIII in Fig. S1) included more than half of the studied accessions.

**Figure 3:**
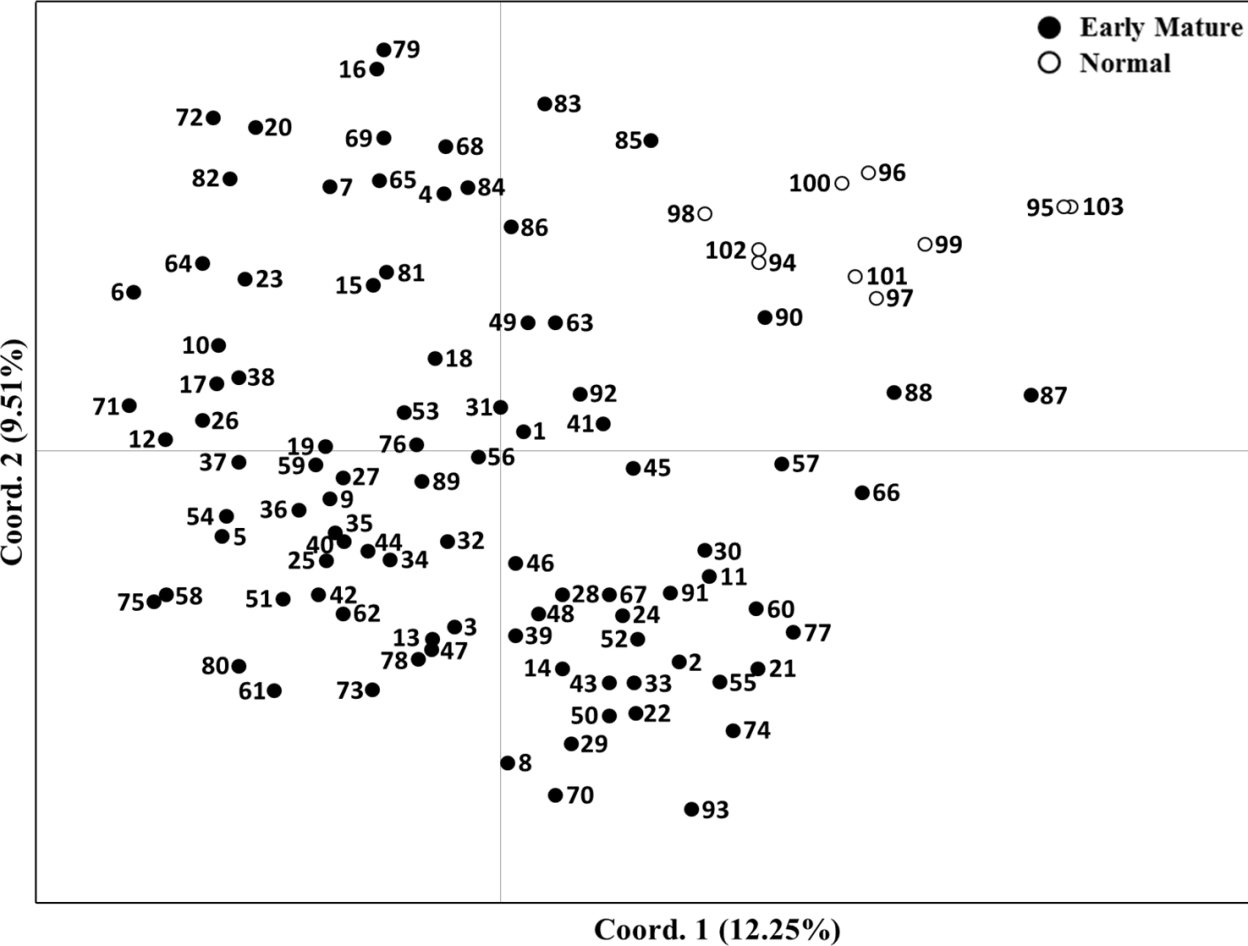
Two-dimensional plot of 103 *Juglans regia* individuals based on two main principal coordinates. Numbers 1–93 are “Early Mature” and numbers 94–103 are “Normal Growth” samples.

Principal coordinate analysis was used to characterize the examined *J. regia* subgroups. The first two principal coordinates accounted for 12.25 and 9.51% of total genetic variation among germplasm. A two-dimensional scatter plot separated the 103 total accessions into two distinct groups and entirely separated “Early Mature” samples from “Normal Growth” walnut samples (Fig. 3).

AMOVA analysis revealed that differentiation among groups was 18%, while differentiation among individuals within groups was 7%. The remaining 75% of variation was within individuals (Table 4). The Mantel test revealed no significant correlation between SSR similarity matrices and morphological traits (*r* = 0.307).

**Table 4:** Analysis of molecular variance based on SSR markers for two groups of *Juglans regia*.

Source of variation	DF	SS	MS	PV	*P*-value
Among groups	1	27.22	27.22	18	<0.001
Among individuals within groups	101	329.98	3.27	7	<0.001
Within individuals	103	281	2.73	75	<0.001
Total	205	638.199		100	

**Notes.**

DF degrees of freedom SS sum of squares MS mean square PV percent variationa

**Figure 4:**
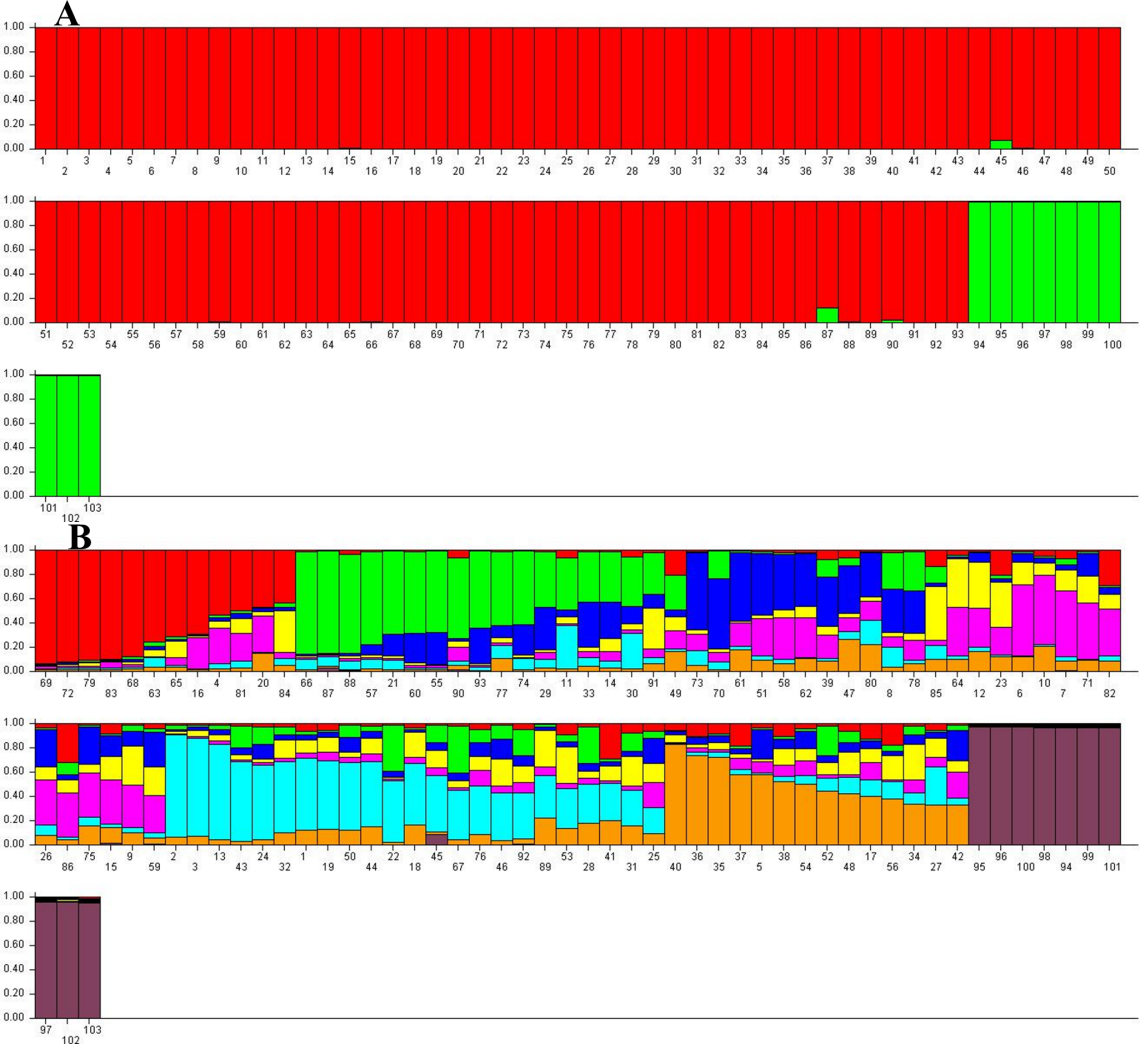
Germplasm structure inference for 103 *J. regia* accessions based on SSR markers obtained by Bayesian assignment using STRUCTURE software for *K* = 2 (A) and *K* = 8 (B). K1, red; K2, green. Numbers 1–93 “Early Mature”, 94–103 “Normal Growth”.

STUCTURE software analyzed the population structure of 103 *J. regia* samples followed by STRUCTURE HARVESTER analyses to obtain the optimum germplasm numbers. A clear peak at *K* = 2 was pinpointed, indicating the 103 samples of Persian walnut could be classified into two main groups (Fig. 4). Akin to the NJ cluster (Fig. S1), all “Early Mature” walnut samples grouped together in the first cluster and “Normal Growth” accessions assembled in the second group. A few admixed samples belonging to the “Early Mature” group were identified by this method. In addition, STRUCTURE HARVESTER produced the second clear peak at *K* = 8. Based on *K* = 8, eight main clusters were identified that each contained several admixed accessions (Fig. 4).

## Discussion

Accelerated growth and development reduces time commitments and costs for breeders and producers (Dierig et al., 2001). Early selection of plant material is vital for expediting breeding program efforts with different tree species (Rweyongeza, Yeh & Dhir, 2004). It is imperative that genetic gain outpace losses in genetic diversity within plantations and open system breeding programs (Diao et al., 2016).

### Phenotypic variation

In studying phenotypic variation among some half-sib walnut seedlings, Rezaee, Vahdati & Valizadeh (2009) reported greater variation in seedling height compared with other traits such as number of nodes and seedling diameter. Previous research by Ambika, Manonmani & Somasundaram (2014) indicated that seed size can influence plant growth characteristics but there was no significant correlation between these two characteristics in our study (Table S2). Wright (1988) noted node numbers during the transition from vegetative growth to the reproductive phase showed a significant positive correlation. Our research reported a significant positive correlation between number of nodes, nut number (*r* = 0.73), and nut weight (*r* = 0.35) in “Early Mature” walnut. Several “Early Mature” accessions showed promise for utilization in breeding programs or as parent material for scion production.

### Genetic diversity

SSR markers are often used to detect variability in Persian walnut accessions. Relatively high levels of polymorphism were observed across *J. regia* accessions in the present study. Mean N_A_ was 6.89 per locus, which was lower than our previous work with the same primer set and similar numbers of accessions (11.5 alleles) (Ebrahimi et al., 2016). The lower allele numbers observed in our experiment could be attributed to the bias toward the specific walnut types used in our study. Genetic diversity can be affected by several factors such as breeding systems, genetic drift, population size, seed dispersal, gene flow, evolutionary history, and natural selection (Hamrick & Godt, 1990). The N_A_ in “Early Mature” seedlings (5 alleles) were higher than “Normal Growth” walnuts (3.88 alleles). Also, *I*, an indicator of genetic diversity, was relatively high and “Early Mature” walnut samples (*I* = 1.28) showed higher values than “Normal Growth” types (*I* = 1.15). Although the presented allelic observations could be attributed to the higher number of “Early Mature” walnuts compared to normal types in our studies, we cannot disregard the increased genetic diversity in this walnut variety.

Our A_E_ numbers were lower than N_A_ in all loci, an indication that not all alleles contributed to diversity. Similar observations were made in previous SSR studies of walnut trees (Pop et al., 2013; Pollegioni et al., 2014; Vahdati et al., 2015; Ebrahimi et al., 2016; Ebrahimi et al., 2017). Among the loci evaluated, only WGA9 had PIC values below 0.50, indicating the studied loci are informative and appropriate for genetic diversity studies. High H_O_ and H_E_ values indicated that these accessions are highly heterozygous. Contrary to *I*, “Normal Growth” walnuts had slightly higher H_O_ than precocious ones. Worthy of note, is that some “Early Mature” seedlings used in our study were likely half-sibs and originated from the same parents, a potential explanation for lower H_O_ values. Alleles showing frequencies below 0.05 were considered rare alleles thus, 65% of alleles had frequencies below 5%. Higher rare allele frequencies were also reported in a previous study of walnut trees (Ebrahimi et al., 2016).

*J. regia* is a heterodichogamous species. Heterodichogamy, an adaptation that facilitates outcrossing (Watanabe, Noma & Nishida, 2016), ultimately improves genetic diversity and could result in alleles with low germplasm frequencies. Outcrossing species usually have considerably higher levels of genetic diversity (Nybom, 2004). The “Early Mature” samples in our study were propagated from seeds, as is standard for the vast majority of existing *J. regia* germplasm in Iran (Ebrahimi et al., 2016). Thus, high levels of rare and private alleles can be attributed to seed propagation and the allogamous nature of *J. regia*.

A high level of private alleles was observed for “Early Mature” and “Normal Growth” walnut samples. Most were frequent although a few (seven alleles in “Early Mature” and one allele in “Normal Growth”) were considered rare alleles in these accessions. These results indicate, for the SSR loci studied, “Early Mature” walnuts are significantly different from “Normal Growth” types. WGA32 and WGA202 loci, which produced the highest N_A_ in the studied loci, also showed the highest number of private alleles (six alleles) in “Early Mature” walnut samples. Further, WGA202 had the highest number of private allele for normal walnuts. Therefore, these two loci may be suitable for discrimination between “Early Mature” and “Normal Growth” walnut samples at either the seed or juvenile phase in breeding programs. However, to confirm these observations, supplementary studies using equivalent samples of these two types of walnut are necessary.

### Genetic structure

Traditional cluster analyses provide an easy and effective way to evaluate the genetic diversity of different collections (Belamkar et al., 2011). Several other statistical approaches, such as population structure (Pritchard, Stephens & Donnelly, 2000) and PCA (Peakall & Smouse, 2012), have been developed and widely used in plant genetics studies (Pollegioni et al., 2014; Ren et al., 2014; Ebrahimi et al., 2016).

A neighbor-joining tree of 103 individuals based on Nei’s genetic distances, showed significant differences between “Early Mature” and “Normal Growth” walnut samples. According to cluster analyses, “Normal Growth” walnuts formed a distinct group and were separated from the “Early Mature” group, indicating that these groups were genetically distinct.

Genetic structure analyses showed SSR markers formed a clear separation among the 103 *J. regia* individuals. This indicates strong differentiation between “Early Mature” and “Normal Growth” walnuts, a conclusion supported by AMOVA analyses. Although the *J. regia* samples we used in our study were not a true population, we performed AMOVA using the two walnut types as groups. AMOVA results indicated a high percentage of variation (18%) existed between the two walnut types. Previous research reports noted molecular variance among different populations ranged from 7% to 13% in *J. regia* germplasms (Christopoulos et al., 2010; Aradhya, Woeste & Velasco, 2010; Vahdati et al., 2015; Ebrahimi et al., 2016). Population variance is almost akin to genetic distances (Ren et al., 2014). The high value of differences between groups compared with the previous works might be due to the high differentiation between “Early Mature” and “Normal Growth” walnuts.

Differentiation into of two groups was supported by the PCA, confirming high genetic differentiation between “Early Mature” and “Normal Growth” accessions. Hence, the clustering analysis by two classification methods revealed a high level of similarity in accession grouping. STRUCTURE analysis confirmed results from the other two clustering methods. The second-best number of sub-clusters observed by STRUCTURE HARVESTER (*K* = 8) corresponded with numbers obtained with the NJ cluster, indicating the two clustering methods could be validated. Therefore, “Early Mature” accessions are genetically different from “Normal Growth” group. Additionally, high admixture was observed in members of the eight sub-clusters. However, members of some sub-clusters were slightly different in each clustering method.

## Conclusion

Our results indicated “Early Mature” walnuts are exhibit relatively high levels of genetic diversity. Further, we noted SSR molecular markers were useful tools for discrimination between “Early Mature” and “Normal Growth” accessions of *J. regia*. Several isolated SSR markers generated “Early Mature”-specific alleles, which could be used to identify these walnut types. Private allele numbers were relatively high, with some alleles considered rare alleles. Rare alleles indicate the distinctness of “Early Mature” walnut samples within local germplasms, and should be a consideration for germplasm conservation. This work indicated employment of SSR primers can facilitate prompt detection of genetically diverse “Early Mature” walnut accessions in seeds and seedlings in breeding programs.

##  Supplemental Information

10.7717/peerj.3834/supp-1 Data S1 Raw data

10.7717/peerj.3834/supp-2 Table S1 Select characteristics of “Early Mature” *Juglans regia* in the third year following seed cultivation SD, seedling diameter (mm); SH, seedling height (cm); NNo, number of nodes; IL, internode length (cm); NNu, number of nuts; NW, average nut weight (g).

10.7717/peerj.3834/supp-3 Table S2 Simple correlation among studied characteristics in “Early Mature” walnut accessions. ^∗∗^*p* < 0.01; ^∗^*p* < 0.05 SD, seedling diameter (mm); SH, seedling height (cm); NNo, number of nodes; IL, internode length (cm); NNu, number of nuts; NW, average nut weight (g).

10.7717/peerj.3834/supp-4 Figure S1 NJ cluster of 103 *Juglans regia* accessions using nine SSR loci. Numbers 1–93 are “Early Mature” samples based on Table 1 and numbers 94–103 are “Normal Growth” samples

## Additional Information and Declarations

### Data Availability

The following information was supplied regarding data availability: Figshare: https://figshare.com/s/9c6623d413fca9bffcf4.
