# Supplementary material for: Evaluation of genetic variability among “Early Mature” Juglans regia using microsatellite markers and morphological traits
Source: PeerJ. 2017 Oct 26;5:e3834. doi: 10.7717/peerj.3834 (PMC5660874; doi:10.7717/peerj.3834)
Supplement: Table S1 — SD, seedling diameter (mm); SH, seedling height (cm); NNo, number of nodes; IL, internode length (cm); NNu, number of nuts; NW, average nut weight (g). [file peerj-05-3834-s002.docx]

| **ID** | SD | SH | NNo | IL | NNu | NW | **ID** | SD | SH | NNo | IL | NNu | NW |
| --- | --- | --- | --- | --- | --- | --- | --- | --- | --- | --- | --- | --- | --- |
| **1** | 71 | 100 | 26 | 7 | 10 | 8.62 | **48** | 21 | 100 | 10 | 8 | 3 | 5.63 |
| **2** | 59 | 80 | 16 | 10 | 9 | 9.33 | **49** | 42 | 100 | 17 | 8 | 4 | 6.86 |
| **3** | 67 | 80 | 6 | 9 | 3 | 9.08 | **50** | 45 | 200 | 27 | 7 | 24 | 11.02 |
| **4** | 61 | 60 | 22 | 7 | 16 | 10.52 | **51** | 46 | 200 | 35 | 7 | 29 | 9.61 |
| **5** | 64 | 150 | 16 | 8 | 10 | 8.76 | **52** | 36 | 100 | 0 | 7 | 1 | 8.11 |
| **6** | 41 | 30 | 10 | 7 | 7 | 6.9 | **53** | 53 | 200 | 11 | 9 | 8 | 8.11 |
| **7** | 52 | 140 | 16 | 7 | 6 | 7.9 | **54** | 25 | 20 | 13 | 9 | 4 | 6 |
| **8** | 46 | 80 | 10 | 8 | 5 | 6.03 | **55** | 25 | 10 | 10 | 9 | 8 | 4.4 |
| **9** | 41 | 90 | 15 | 5 | 4 | 5.74 | **56** | 44 | 200 | 11 | 9 | 6 | 6.95 |
| **10** | 62 | 100 | 39 | 5 | 18 | 10.82 | **57** | 26 | 10 | 16 | 7 | 10 | 8.83 |
| **11** | 45 | 180 | 17 | 10 | 14 | 6.35 | **58** | 51 | 150 | 12 | 21 | 5 | 7.96 |
| **12** | 36 | 100 | 0 | 0 | 1 | 6.82 | **59** | 51 | 20 | 10 | 10 | 2 | 9.57 |
| **13** | 71 | 100 | 22 | 10 | 20 | 7.28 | **60** | 63 | 240 | 21 | 6 | 4 | 11.11 |
| **14** | 13 | 36 | 0 | 0 | 1 | 5 | **61** | 53 | 150 | 20 | 7 | 6 | 5.26 |
| **15** | 72 | 150 | 20 | 7 | 13 | 10.01 | **62** | 54 | 100 | 9 | 9 | 3 | 9.33 |
| **16** | 15 | 70 | 0 | 0 | 4 | 3.6 | **63** | 55 | 30 | 12 | 8 | 10 | 4.84 |
| **17** | 18 | 38 | 0 | 0 | 5 | 4.7 | **64** | 65 | 100 | 18 | 11 | 12 | 9.74 |
| **18** | 34 | 7 | 14 | 11 | 10 | 7.47 | **65** | 53 | 200 | 7 | 8 | 3 | 8.86 |
| **19** | 22 | 190 | 21 | 7 | 8 | 10.32 | **66** | 43 | 8 | 17 | 7 | 12 | 7.64 |
| **20** | 37 | 20 | 22 | 8 | 22 | 8.35 | **67** | 50 | 180 | 35 | 8 | 3 | 6.5 |
| **21** | 39 | 80 | 9 | 8 | 3 | 8.13 | **68** | 52 | 100 | 27 | 10 | 19 | 5.87 |
| **22** | 12 | 15 | 0 | 0 | 1 | 5.7 | **69** | 53 | 100 | 24 | 8 | 15 | 5.85 |
| **23** | 55 | 80 | 16 | 6 | 4 | 7.11 | **70** | 46 | 110 | 13 | 5 | 10 | 5.7 |
| **24** | 37 | 10 | 14 | 6 | 6 | 8.8 | **71** | 49 | 80 | 17 | 7 | 17 | 4.62 |
| **25** | 46 | 80 | 18 | 10 | 5 | 7.62 | **72** | 53 | 80 | 0 | 7 | 1 | 5.83 |
| **26** | 48 | 110 | 40 | 6 | 18 | 5.82 | **73** | 44 | 80 | 35 | 9 | 6 | 5.28 |
| **27** | 36 | 10 | 21 | 6 | 11 | 4.16 | **74** | 39 | 10 | 18 | 10 | 5 | 6.48 |
| **28** | 13 | 10 | 0 | 0 | 1 | 1.7 | **75** | 43 | 30 | 19 | 10 | 5 | 8 |
| **29** | 28 | 47 | 5 | 4 | 2 | 3.67 | **76** | 23 | 30 | 23 | 5 | 11 | 9.03 |
| **30** | 36 | 130 | 24 | 4 | 12 | 3.47 | **77** | 18 | 160 | 10 | 8 | 3 | 4.17 |
| **31** | 37 | 114 | 14 | 8 | 11 | 4.56 | **78** | 17 | 150 | 15 | 8 | 2 | 7.33 |
| **32** | 36 | 7 | 7 | 9 | 3 | 3.24 | **79** | 14 | 30 | 16 | 9 | 12 | 8.13 |
| **33** | 22 | 27 | 7 | 8 | 4 | 6.57 | **80** | 52 | 30 | 34 | 8 | 15 | 12.11 |
| **34** | 53 | 95 | 25 | 5 | 8 | 7.82 | **81** | 38 | 40 | 25 | 8 | 18 | 10.41 |
| **35** | 53 | 100 | 11 | 9 | 4 | 7.51 | **82** | 35 | 200 | 12 | 7 | 9 | 5.04 |
| **36** | 47 | 150 | 11 | 8 | 6 | 10 | **83** | 48 | 30 | 25 | 7 | 17 | 7.8 |
| **37** | 44 | 15 | 34 | 5 | 29 | 8.77 | **84** | 26 | 80 | 8 | 8 | 8 | 6.17 |
| **38** | 47 | 200 | 10 | 7 | 3 | 8.48 | **85** | 34 | 80 | 17 | 7 | 6 | 5.47 |
| **39** | 62 | 100 | 17 | 8 | 12 | 10.87 | **86** | 32 | 30 | 18 | 8 | 7 | 7.21 |
| **40** | 17 | 15 | 0 | 0 | 1 | 10.25 | **87** | 41 | 30 | 25 | 7 | 17 | 6.79 |
| **41** | 69 | 100 | 14 | 8 | 11 | 6.14 | **88** | 28 | 130 | 8 | 9 | 6 | 7.65 |
| **42** | 57 | 100 | 16 | 6 | 10 | 8.83 | **89** | 33 | 30 | 25 | 6 | 24 | 6.15 |
| **43** | 26 | 35 | 10 | 6 | 8 | 7.55 | **90** | 50 | 15 | 22 | 9 | 20 | 6.83 |
| **44** | 53 | 100 | 17 | 9 | 13 | 11.09 | **91** | 46 | 30 | 20 | 2 | 12 | 11.98 |
| **45** | 71 | 200 | 30 | 8 | 27 | 14.42 | **92** | 55 | 30 | 20 | 0.09 | 15 | 9.51 |
| **46** | 57 | 150 | 28 | 9 | 21 | 11.03 | **93** | 36 | 5 | 17 | 0.03 | 12 | 12.48 |
| **47** | 57 | 100 | 18 | 8 | 12 | 7.5 |  |  |  |  |  |  |  |
