# Supplementary material for: Evaluation of genetic variability among “Early Mature” Juglans regia using microsatellite markers and morphological traits
Source: PeerJ. 2017 Oct 26;5:e3834. doi: 10.7717/peerj.3834 (PMC5660874; doi:10.7717/peerj.3834)
Supplement: Table S2 — SD, seedling diameter (mm); SH, seedling height (cm); NNo, number of nodes; IL, internode length (cm); NNu, number of nuts; NW, average nut weight (g). [file peerj-05-3834-s003.docx]

|  | SD | SH | NNo | NNu | IL | NW |
| --- | --- | --- | --- | --- | --- | --- |
| TD | 1 |  |  |  |  |  |
| SH | 0.358** | 1 |  |  |  |  |
| Nno | 0.444** | 0.168 | 1 |  |  |  |
| NNu | 0.365** | 0.054 | 0.728** | 1 |  |  |
| IN | 0.366** | 0.245* | 0.212* | 0.089 | 1 |  |
| NW | 0.446** | 0.197 | 0.353** | 0.357** | 0.106 | 1 |
