# Supplementary figures and images for: Evaluation of genetic variability among “Early Mature” Juglans regia using microsatellite markers and morphological traits
Source: PeerJ. 2017 Oct 26;5:e3834. doi: 10.7717/peerj.3834 (PMC5660874; doi:10.7717/peerj.3834)

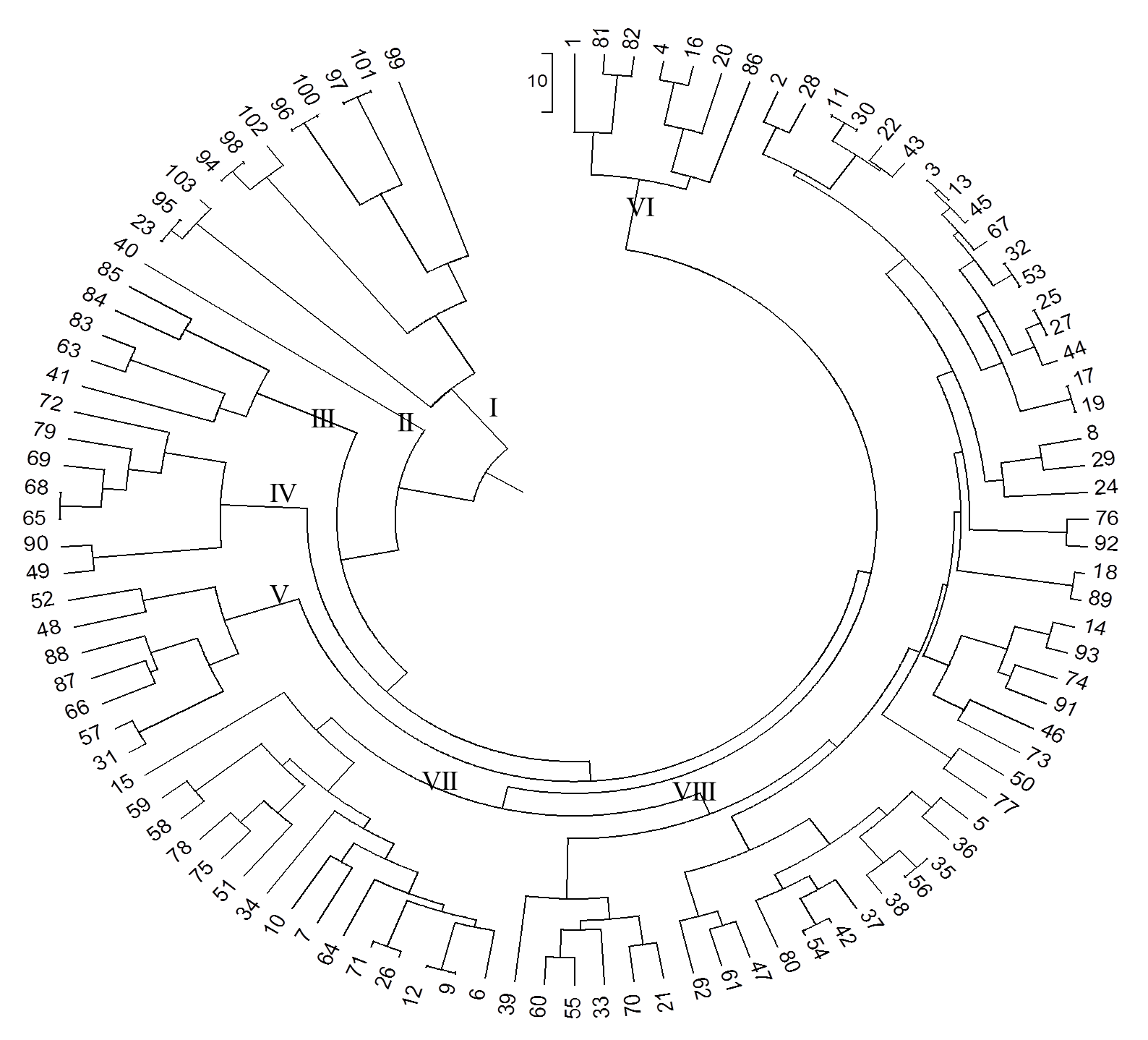

Supplement: Figure S1 [file peerj-05-3834-s004.png]
